# Society of Alumni, University of Pennsylvania

**Published:** 1894-08

**Authors:** 


### SOCIETY OF ALUMNI, UNIVERSITY OF PENNSYLVANIA.

The Fourteenth Annual Meeting of the Society of the Alumni, Department of Dentistry, University of Pennsylvania, took place at the Colonnade Hotel, Philadelphia, the afternoon of June 6, 1894, Dr. Solomon Freeman, of New York, presiding. Members were present from New York, New Jersey, Delaware, Massachusetts, and Connecticut, as well as from the interior of this State. These, with the members residing in the city, numbered thirty-five.

Dr. Edw. H. Allen, of Freeport, Ill., presented a paper, which was read by Dr. McFadden, of this city, entitled "To what Extent should a Dentist be Conservative." Dr. A. H. Porter, of Germantown, gave a *résumé* of a lengthy paper on "The Physiology of Hypnotism," which will be published in the INTERNATIONAL DENTAL JOURNAL and *Alumni Annual*, the official organ of the Society.

The officers elected for the ensuing year are W. L. Winner, D.D.S., Philadelphia, President; F. A. Peeso, D.D.S., W. E. Christensen, D.D.S., and A. H. Porter, D.D.S., Vice-Presidents; J. Edw. Dunwoody, Philadelphia, Recording Secretary and Treasurer; George C. Küsel, M.D., D.D.S., Corresponding Secretary; H. B. McFadden, D.D.S., and George T. Cornelius, D.D.S., members of Executive Committee.

